# Two Modes of Riboflavin-Mediated Extracellular Electron Transfer in *Geobacter uraniireducens*

**DOI:** 10.3389/fmicb.2018.02886

**Published:** 2018-11-27

**Authors:** Lingyan Huang, Jiahuan Tang, Man Chen, Xing Liu, Shungui Zhou

**Affiliations:** Fujian Provincial Key Laboratory of Soil Environmental Health and Regulation, College of Resources and Environment, Fujian Agriculture and Forestry University, Fuzhou, China

**Keywords:** *Geobacter uraniiireducens*, flavins, electron shuttles, extracellular electron transfer, cytochromes bound cofactor

## Abstract

Anaerobes respire extracellular electron acceptors by extracellular electron transfer (EET). It is widely recognized that flavins can act as electron shuttles to facilitate this process. Flavin synthesis genes are widely distributed in *Geobacter* species. However, the functions of flavins in the EET of *Geobacter* species are unclear. Here, we demonstrate that *G. uraniireducens* can secrete abundant riboflavin (up to 270 nM) to facilitate EET. When an electrode was used as the electron acceptor, the quick recovery of anodizing current after anolyte replacement and the electrochemical behavior of the *G. uraniireducens* biofilm characterized by differential pulse voltammetry suggest that the self-secreted riboflavin promoted EET by serving as bound redox cofactors for cytochromes. On the contrary, when Fe(III) oxide was the electron acceptor, free riboflavin acted as electron shuttle to mediate the reduction of Fe(III) oxide. The results demonstrate the flexibility of flavins in EET, suggesting that the properties of electron acceptors can affect the binding mode of extracellular flavins, and broaden the knowledge of the EET of *Geobacter* species.

## Introduction

Anaerobic organisms can respire extracellular electron acceptors to conserve energy for growth by extracellular electron transfer (EET). This process affects the cycling of minerals, promotes the degradation of organic and radioactive wastes, helps current generation in the microbial fuel cell and contributes to the electron exchange between some syntrophic species (Nealson and Saffarini, 1994; Lovley, 2006, 2011; Nevin et al., 2010; Liu et al., 2018). Three EET methods have been reported (Newman and Kolter, 2000; Shi et al., 2016; Kumar et al., 2017): (i) cytochromes mediate direct electron transport after intimate contact between microbes and terminal electron acceptors; (ii) conductive pili mediate long-range electron transfer for the reduction of remote electron acceptors; and (iii) electron shuttles mediate extracellular electron transport. Notably, the specific methods used are species dependent.

Species from the genus of *Shewanella* and *Geobacter* are widely used for research on EET. *Shewanella* species can excrete abundant free flavins which are supposed to act as electron shuttles for the reduction of extracellular electron acceptors (Marsili et al., 2008). Recent studies further indicated that physiological concentrations of flavins mainly bind to outer membrane cytochromes with a high dissociation constant and can be cofactors mediating extracellular electron transport in *Shewanella* species (Okamoto et al., 2013, 2014a). In contrast, *Geobacter* species have abundant c-type cytochromes and are generally thought to transfer electrons to terminal electron acceptors by direct contact (Bond and Lovley, 2003; Reguera et al., 2005). Surprisingly, flavin synthesis and excretion genes are widely distributed in *Geobacter* species, which indicates that flavins may widely participate in the EET of *Geobacter* species. Recent studies also indicated that *Geobacter sulfurreducens* can uptake self-secreted flavins as bound cofactors for EET (Okamoto et al., 2014c; Michelson et al., 2017). However, the detailed functions of flavins in the EET of *Geobacter* species have not been documented and the possibility that free flavin molecules act as electron shuttles in the EET of *Geobacter* species has not been reported.

*Geobacter uraniireducens* is deficient in current generation but is a good iron oxide reducer, even when it is not in direct contact with iron oxide (Rotaru et al., 2015). In most *Geobacter* species, conductive pili are necessary for the efficient reduction of extracellular electron acceptors (Morita et al., 2011; Malvankar and Lovley, 2014; Rotaru et al., 2014). However, the pili of *G. uraniireducens* are nonconductive (Tan et al., 2016). Considering there are integral flavin synthesis genes coordinating 2539702 to 2543699 in the genome of *G. uraniireducens* (accession no. NC_009483.1), we suppose that self-secreted flavins participate in the EET of *G. uraniireducens*. In this study, abundant self-secreted flavins from *G. uraniireducens* were identified using fluorescence spectrophotometry and liquid chromatography-electrospray ionization ion-trap mass spectrometry. The possibility of flavins acting as free electron shuttles in Fe(III) oxide reduction was verified. Differential pulse voltammetry was used to reveal the redox properties of self-secreted flavins and to identify the role of flavins in anode respiration for current generation.

## Materials and Methods

### Bacterial Strains and Growth Conditions

*G. uraniireducens* strain Rf4 and *G. sulfurreducens* strain PCA were inoculated from frozen stocks in our lab and were cultured at 30°C under strict anaerobic conditions in NBAF medium as previously reported (Coppi et al., 2001).

### Fluorescence Spectrophotometry

To identify and quantify the secreted flavins, cell cultures from *G. uraniireducens* Rf4 and *G. sulfurreducens* PCA were collected and filtered through 0.22-μm membrane filters. Fluorescence spectra of the filtrates were recorded using the Agilent Cary Eclipse Fluorescence Spectrometer (Agilent Technologies, California, United States) as previously described (Okamoto et al., 2014c). Emission spectra were measured at an excitation of 440 nm, and excitation spectra were monitored at 520 nm. A standard curve from a gradient concentration of riboflavin (0, 100, 300, and 500 nM) was calculated to quantify the amount of free flavins in the culture medium.

### Liquid Chromatography-Electrospray Iron-Trap Mass Spectrometry

Liquid chromatography (Agilent 1100, United States)-electrospray ion-trap mass spectrometry (Agilent MSD-Trap-XCT, United States) (LC/ESI-MS) was performed to detect the secretion of flavins by *G. uraniireducens*. A total of 10 μL of filtered culture was subjected to LC/ESI-MS analysis at a flow rate of 1 mL min^−1^. Flavins were separated by a reversed-phase C_18_ column (Eclipse Plus C_18_, 4.6 mm × 250 mm, 5 μm) and detected with the UV-Vis detector setting at 468/525 nm (excitation/emission). An acetic acid/methanol gradient was used to elute flavins as previously reported(You et al., 2018), with the mobile phase comprising 650 mM glacial acetic acid (phase A) and methanol (phase B). Riboflavin and flavin mononucleotides were purchased from Sigma and served as reference standards.

### Fe (III) Oxide Reduction

Ferrihydrite beads (3∼5 mm diameter) with a molecular mass cutoff of 12 kDa were prepared as described previously (Nevin and Lovley, 2000). There was ca. 10% of ferrihydrite, which can be reduced by microorganisms directly, exposed on their surfaces. To prepare the Fe(III) oxide growth medium, free ferrihydrite or ferrihydrite beads were added with a final concentration of Fe(III) of approximately 50 mM. Acetate (10 mM) was supplied as an electron donor. The same amount of mid-exponential phase cells growing in NBAF medium were collected and washed with PBS and then were used as inocula. The production of Fe(II) was measured at different time points with the Ferrozine assay as previous reported (Lovley and Phillips, 1988).

### Flavin Reduction Assay

Flavins are fluorescent in an oxidized state (Coursolle et al., 2010). To test the reduction of riboflavin *in vivo* by *G. uraniireducens*, 3 mL culture was collected, washed once with fresh medium, and then injected into an anaerobic cuvette containing 300 nM of riboflavin. The reduction of riboflavin was monitored by a fluorescence spectrophotometer at different time points as described above. Subsequently, the cuvette was exposed to the atmosphere, vortexed for approximately 10 s to reoxidize the riboflavin, and then resealed and tested for fluorescence again.

### Reduction of Ferrihydrite by Riboflavin

Riboflavin was reduced as previously described using hydrogen as the reductant in the presence of palladium (Von Canstein et al., 2008). 0.1 mM of reduced riboflavin was added into the medium containing 5 mM ferrihydrite and incubated for 24 h in the dark at room temperature. The reduction of Fe(III) was determined after testing the production of Fe(II) by the ferrozine assay (Lovley and Phillips, 1988).

### SDS-PAGE and Heme-Staining

Extracellular proteins in anode biofilm matrix were collected as previously described (Sun et al., 2012). Extracellular proteins were separated from ferrihydrite culture medium as described previously (Smith et al., 2014). Briefly, 200 mL of ferrihydrite culture were shacked heavily and then centrifuged at 10000 × *g* for 20 min. Supernatants were further pass through a 0.22-mm filter to remove residue cells and ferrihydrite. Extracellular proteins were concentrated using Amicon Centrifugal filter (3 kDa cutoff, Merk millipore) and quantify with the Micro BCA protein assay kit (Thermo Fisher Scientific). Proteins were mixed with 5 × non-reducing loading dye and then 5 μg proteins were loaded on a 12.5% Tris-tricine polyacrylamide gel. The cytochromes were heme stained in the gel with N,N,N′,N′-tetramethylbenzidine (Liu et al., 2014).

### Fuel Cell Construction and Electrochemical Measurements

A single-chamber, three-electrode system with a liquid volume of 20 mL and a headspace volume of 10 mL was constructed for the electrochemical measurement as previously described (Okamoto et al., 2013). An indium tin-oxide glass (4 cm × 5 cm) was used as a working electrode and placed at the bottom of the reactor. Hg| Hg_2_Cl_2_ (sat. KCl) and a graphite plate (1.5 cm × 1 cm × 0.5 cm) were used as the reference and counter electrode, respectively. The electrolyte was freshwater medium (Bond and Lovley, 2003) supplied with 10 mM acetate. A total of 2 mL of NBAF cultures growing to the mid-exponential phase were collected and washed before cell inoculation.

Current production was monitored with a 1000C electrochemical workstation (CH instruments, United States) with a work electrode polarized at +0.3 V (vs. SCE). Differential pulse voltammetry (DPV) was conducted from –800 mv to 200 mV (vs. SCE). The specific parameters were: *E*_i_ = −0.8 V, *E*_f_ = 0.2 V, pulse height 50 mV, pulse width 250 ms, step height 2 mV, step time 500 ms, scan rate 1 mV s^−1^.

## Results and Discussion

### *Geobacter uraniireducens* Secretes Abundant Riboflavin

**FIGURE 1:**
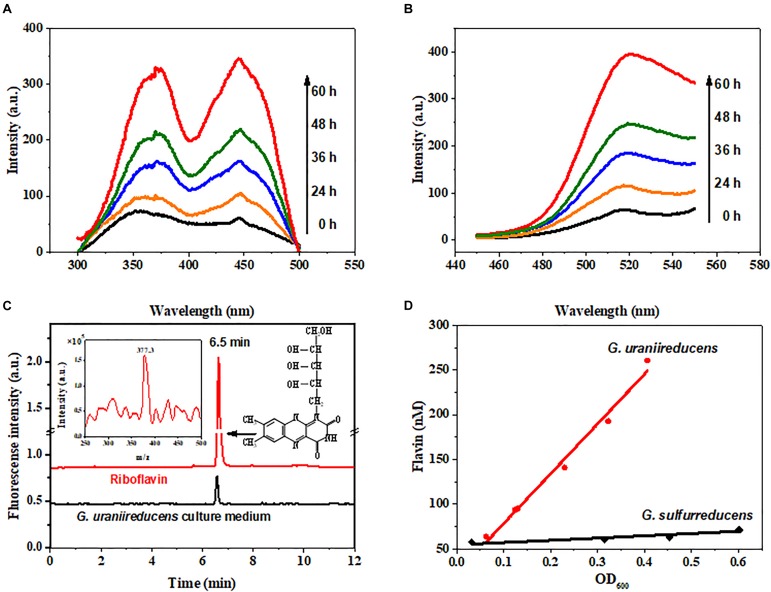
Identification of flavins secreted by *G. uraniireducens*. **(A)** Excitation and **(B)** emission spectra (arbitrary units, a.u.) of the cell-free culture medium collected from the *G. uraniireducens* culture after 0, 24, 36, 48, and 60 h of inoculation. The excitation wavelength was 440 nm and the emission wavelength was 520 nm. **(C)** LC/ESI-MS identification of riboflavin from the cell-free culture medium of *G. uraniireducens* collected at the stationary phase. The fluorescence detector was set at 468/525 nm (excitation/emission). The LC peak at 6.5 min corresponds to the compound of riboflavin with a *m/z* of 377.3, and 0.1 μM riboflavin was used as the reference standard. **(D)** Quantification of accumulated flavins as a function of the cell densities of *G. uraniireducens* and *G. sulfurreducens*.

Fluorescence spectrophotometry was used to identify the redox molecules in the *G. uraniireducens* culture. The fluorescence spectra (Figures 1A,B) of the *G. uraniireducens* culture have a characteristic emission peak at 520 nm under excitation at both 370 nm and 440 nm, identical to the fluorescence spectra of flavins (Supplementary Figure S1). The secretion of flavins was further confirmed by LC/ESI-MS, which showed the characteristics of riboflavin (Figure 1C). There are three forms of flavins inside cells, namely, flavin adenine dinucleotide (FAD), flavin mononucleotide (FMN) and riboflavin (RF). FMN and RF can be involved in EET, but FAD is only released when cells are dead (Von Canstein et al., 2008). The data indicated that RF was the dominant flavin excreted by *G. uraniireducens* (Figure 1C and Supplementary Figure S2). Notably, *G. uraniireducens* excreted a much higher concentration of free riboflavin than *G. sulfurreducens* (with maximums of 270 nM and 70 nM for *G. uraniireducens* and *G. sulfurreducens* at the stationary phase, respectively). In addition, the riboflavin concentrations of both cells were increased with the growth of cells (Figure 1D and Supplementary Figure S3).

### Free Riboflavin Act as Electron Shuttles to Facilitate Fe(III) Oxide Reduction

**FIGURE 2:**
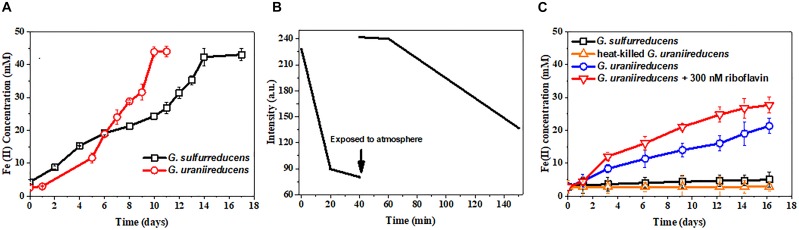
Free riboflavin facilitates the extracellular electron transfer of *G. uraniireducens*. **(A)** Fe(III) oxides reduction by *G. uraniireducens* and *G. sulfurreducens*. **(B)** Riboflavin reduction by *G. uraniireducens*. The arrow indicates the time point at which the culture was exposed to the atmosphere and vortex. The redox state of riboflavin was monitored by fluorescence emission at 520 nm with excitation at 440 nm. **(C)** Ferrihydrite bead reduction. The same number of cells were used as inocula. Data are averages for triplicate cultures.

*Geobacter uraniireducens* can reduce Fe(III) oxide much faster than *G. sulfurreducens* (Figure 2A). Previous studies have implied that conductive pili are necessary for the efficient EET of *Geobacter* species to reduce Fe(III) oxide (Reguera et al., 2005; Liu et al., 2014). However, the pili of *G. uraniireducens* are nonconductive. Additional methods of electron transfer are therefore necessary to contribute to the high Fe(III) oxide reduction of *G. uraniireducens*.

Fluorescence spectrophotometry was also performed on the Fe(III) oxide-grown culture of *G. uraniireducens*. Not surprisingly, riboflavin could be detected in the Fe(III) oxide culture medium of *G. uraniireducens* (Supplementary Figure S4), which is in contrast to the riboflavin-free Fe(III) oxide culture of *G. sulfurreducens* (data not shown) and indicates an EET involving free riboflavin. The redox state of riboflavin can be detected by fluorescence spectrometry as only oxidized riboflavin fluoresces (Ghisla et al., 1974). The possibility that oxidized riboflavin can accept electrons from *G. uraniireducens* was examined with fluorescence spectrophotometry. Oxidized riboflavin was incubated with *G. uraniireducens*. The fluorescence intensity of the riboflavin decreased over time, indicating the continuous reduction of riboflavin by *G. uraniireducens* and the bio-reduced riboflavin could be re-oxidized after exposure to atmospheric oxygen (Figure 2B). Furthermore, 0.18 mM of Fe(II) was generated when 0.1 mM of reduced riboflavin was added into 5 mM ferrihydrite medium indicating that riboflavin can carry electrons for the reduction of Fe(III) oxide. To determine whether the free riboflavin excreted by *G. uraniireducens* accelerated insoluble Fe(III) reduction, ferrihydrite trapped in hydrogel beads was used as an electron acceptor. The insulated hydrogel shell prevented the direct contact between *G. uraniireducens* cells and ferrihydrite. As shown in Figure 2C, *G*. *uraniireducens* readily reduced the ferrihydrite beads, generating Fe(II) with a concentration of approximately 20 mM after 16 days. In contrast, *G. sulfurreducens* reduced less than 5 mM of Fe(III) via the direct reduction of the Fe(III) oxide exposed on the surface of the ferrihydrite beads. The addition of riboflavin further accelerated the reduction of Fe(III) oxide by *G. uraniireducens*, increasing this value from 0.78 mM⋅day^−1^ to 1.54 mM⋅day^−1^.

### Riboflavin Bound as Cofactors to Cytochromes Promote Current Production

**FIGURE 3:**
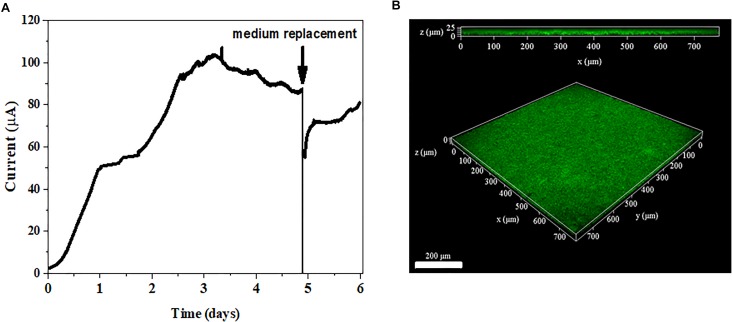
Current generation and anode biofilm formation. **(A)** Current production of *G. uraniireducens*. The arrow indicates the replacement of the electrolyte. **(B)** Confocal scanning fluorescence microscope image of *G. uraniireducens* biofilm growing on the anode. Cells were stained with LIVE/DEAD stain. The scale bar represents 200 μm.

The anode reduction of *G. uraniireducens* was tested in a three-electrode system (Figure 3A). The current was generated continuously. When the plateau arrived, the electrolyte was replaced with fresh medium. The current was immediately recovered to 80% of the original value (Figure 3A). When mediators are involved in EET for current generation, the replacement of the electrolyte with fresh medium disrupts the current production (Marsili et al., 2008). The immediate current recovery after the medium exchange indicated that EET mediated by electron shuttles did not dominate the EET of *G. uraniireducens* when an anode was the electron acceptor. Notably, most of the *G. uraniireducens* were attached on the anode and formed a compact electroactive biofilm (Figure 3B), indicating the possibility of anode reduction with direct contact.

**FIGURE 4:**
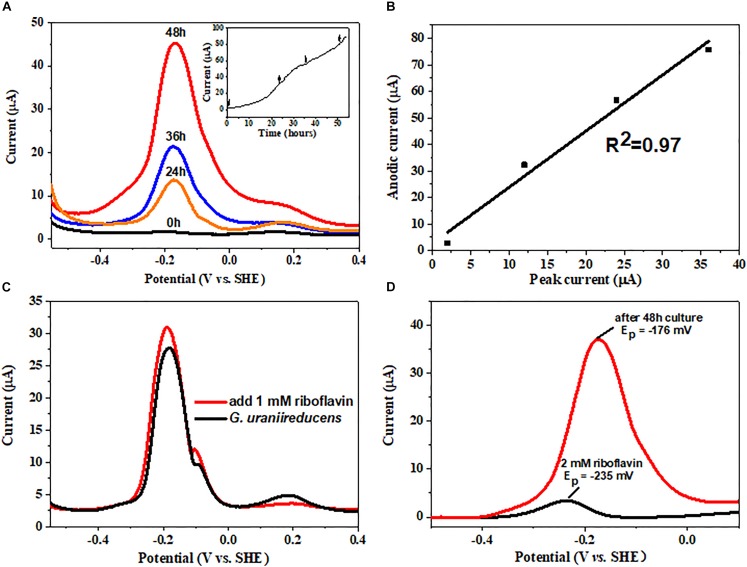
Differential pulse voltammetry (DPV) analysis of the *G. uraniireducens* anode biofilm. **(A)** Differential pulse voltammogram of the *G. uraniireducens* anode 0, 24, 36, and 48 h after inoculation. The inserted figure shows the anodic current profile, with arrows representing the time for DPV analysis. **(B)** Plot of redox peak current at a potential of –176 mV against the corresponding anodic current. **(C)** DPV for biofilms of *G. uraniireducens* before and after the addition of 1 μM riboflavin. **(D)** Base-line subtracted differential pulse voltammogram for 2 μM riboflavin solution and for *G. uraniireducens* anode biofilm after 48 h growth.

The electrochemical behavior of the *G. uraniireducens* biofilm was further characterized. Differential pulse voltammetry (DPV) was conducted at different time points during the process of biofilm formation. Three peaks were detected. Notably, only the redox peak current at −176 mV increased with time (Figure 4A) and was positively correlated with the current produced (Figure 4B). In particular, the addition of riboflavin increased the redox peak current at the same potential (Figure 4C). These data indicate that the redox peak at −176 mV can be assigned to the redox cycling of riboflavin and that the excretion of riboflavin can promote the delivery of electrons produced by *G. uraniireducens* to the anode. However, the redox peak potential of free riboflavin is approximately −235 mV (Figure 4D), which is negative than that of riboflavin in *G. uraniireducens* biofilm. This discrepancy has also been reported in *G. sulfurreducens* and the reason can be contributed to the binding of riboflavin to cytochromes positively shifts the redox peak potential (Tan et al., 2016). Therefore, riboflavin acted as cofactors of cytochromes to promote current production in *G. uraniireducens* when an anode was the electron acceptor.

### Different Outer Membrane Cytochrome Profiles

**FIGURE 5:**
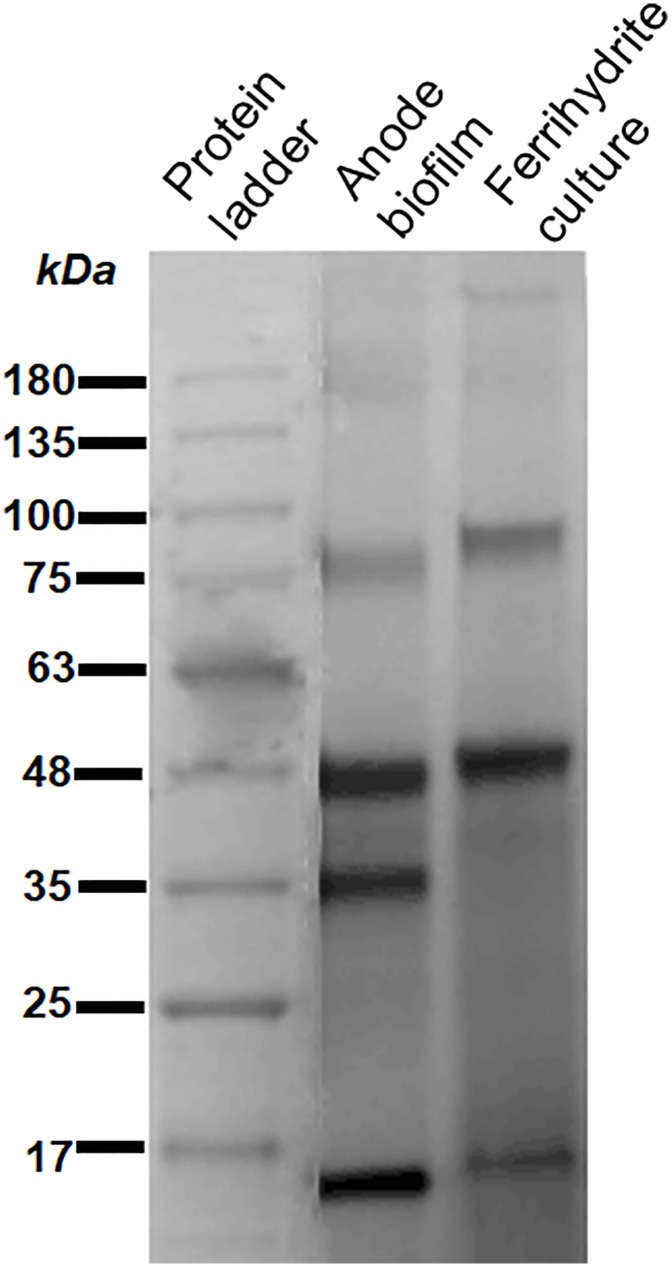
Heme-stained SDS-PAGE gel of outer membrane *c*-type cytochromes extracted from the anode biofilm and ferrihydrite culture medium of *G. uraniireducens*. The protein marker is Biostep Prestained Protein Marker (Tanon, Shanghai, China).

Riboflavin facilitates the electron transfer between extracellular *c*-type cytochromes and electron acceptors. Previous studies have indicated that the binding affinities of flavins to different cytochromes may differ (Okamoto et al., 2014b). When flavins have high binding affinities to cytochromes, they tend to form cofactors; otherwise, they tend to be free. The outer membrane proteins were extracted from *G. uraniireducens* anode biofilm and ferrihydrite culture medium, and were analyzed to identify *c*-type cytochrome profiles. Both the heme-stained SDS-PAGE gel (Figure 5) and mass spectrometry analysis (Supplementary Table S1) indicated a different *c*-type cytochrome distribution between *G. uraniireducens* growing on anode and in ferrihydrite medium. Especially, the *c*-type cytochromes in *G. uraniireducens* anode biofilm showed much more variety (Supplementary Table S1).

### Implication

The results demonstrate that *G. uraniireducens* can excrete abundant riboflavin, which mediates EET to reduce extracellular electron acceptors, suggesting that the existing mode of extracellular riboflavin is dependent on the status of terminal electron acceptors. When Fe(III) oxides acted as the electron acceptor, *G. uraniireducens* used free riboflavin to facilitate EET. In contrast, when the electrode was an electron acceptor, the excreted riboflavin bound to cytochromes acting as cofactors to accelerate EET.

Our studies also indicate that *G. uraniireducens* expresses different outer membrane *c*-type cytochromes when reduces different electron acceptors, and these cytochromes probably have different binding affinity toward riboflavin that cytochromes in anode biofilm show higher binding affinity toward riboflavin than the cytochromes on ferrihydrite reduction cells. However, the different existing modes of extracellular riboflavin can help *G. uraniireducens* to survive in different environments. The free flavins mediate two-electron transfer between cytochromes and electron acceptors, while the bound flavin cofactor facilitates a one-electron redox reaction, which is much more efficient (Okamoto et al., 2013). Fe(III) oxides are consumable, and not all are accessible to *Geobacter* species. Expression of conductive pili can alleviate the stress. However, the pili of *G. uraniireducens* are nonconductive. The excretion of free riboflavin in *G. uraniireducens* facilitates the transfer of electrons to remote or nonaccessible Fe(III) oxides. In contrast, the electrode is a permanent electron acceptor, and the EET in the electroactive biofilm is facilitated by cytochromes. The *G. uraniireducens* biofilm is compact. Flavins binding on cytochromes can greatly accelerate the electron exchange among cytochromes and at the interface between the cytochromes and electrode.

In summary, this study reveals that the existing modes of extracellular flavins in EET are flexible. The direct extracellular electron transfer in *Geobacter* species is well-known. However, the flavin-mediated EET in *Geobacter* species is not generally recognized. This study not only broadens the knowledge of EET in *Geobacter* species but also provides a new perspective to understand EET in natural systems.

## Author Contributions

XL and SZ designed the experiments. LH ran all the tests with the help of JT. MC contributed to the scientific discussion. LH and XL wrote the original manuscript with revisions from all authors.

## Conflict of Interest Statement

The authors declare that the research was conducted in the absence of any commercial or financial relationships that could be construed as a potential conflict of interest.
