# Supplementary material for: Two Modes of Riboflavin-Mediated Extracellular Electron Transfer in Geobacter uraniireducens
Source: Front Microbiol. 2018 Nov 27;9:2886. doi: 10.3389/fmicb.2018.02886 (PMC6277576; doi:10.3389/fmicb.2018.02886)
Supplement: Supplementary file 1 [file Data_Sheet_1.docx]

Supplementary Material

Two modes of riboflavin-mediated extracellular electron transfer in *Geobacter uraniireducens*

Lingyan Huang, Jiahuan Tang, Man Chen, Xing Liu^*^, Shungui Zhou

*** Correspondence:**

Xing Liu

[xingliu@fafu.edu.cn](mailto:xingliu@fafu.edu.cn)


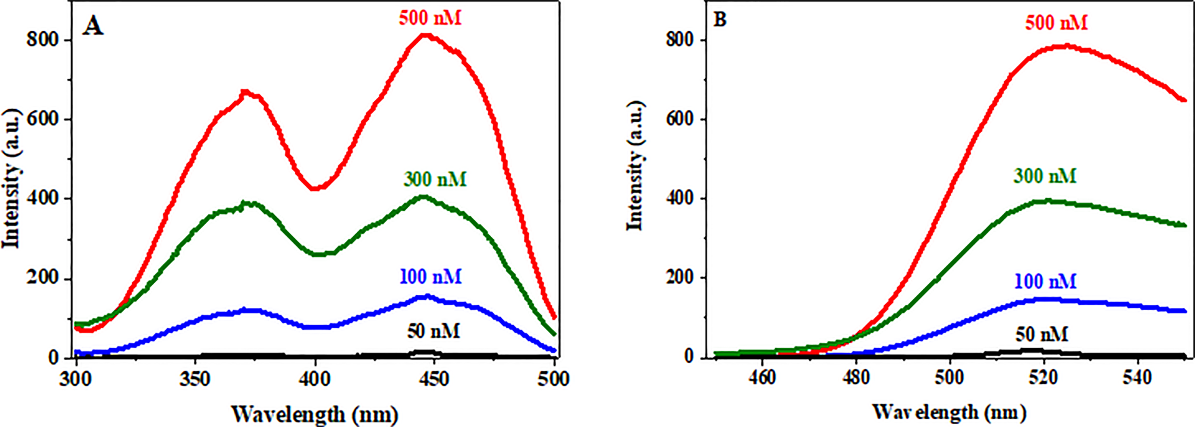


**Figure S1.** Excitation (A) and emission (B) spectra of riboflavin at concentration of 50, 100, 300, 500 nM.

**
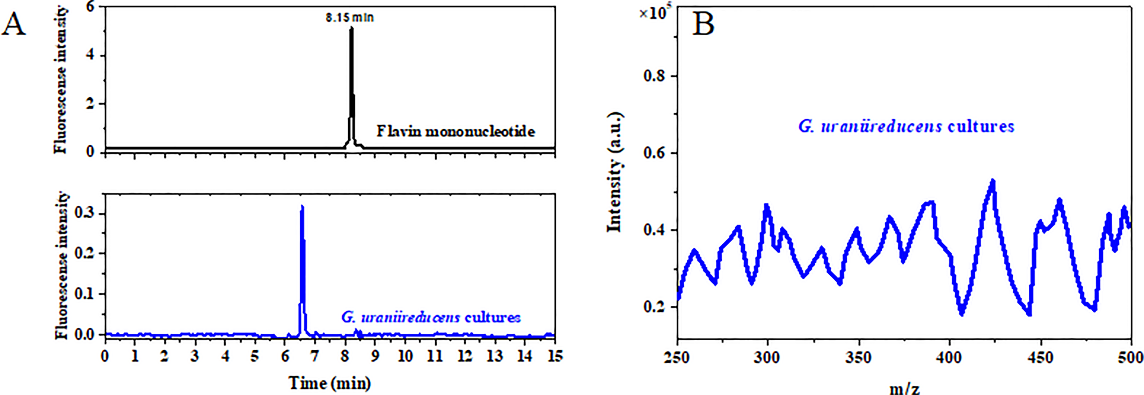
**

**Figure S2.** Liquid chromatography pattern (A) and mass spectrum (B).

**
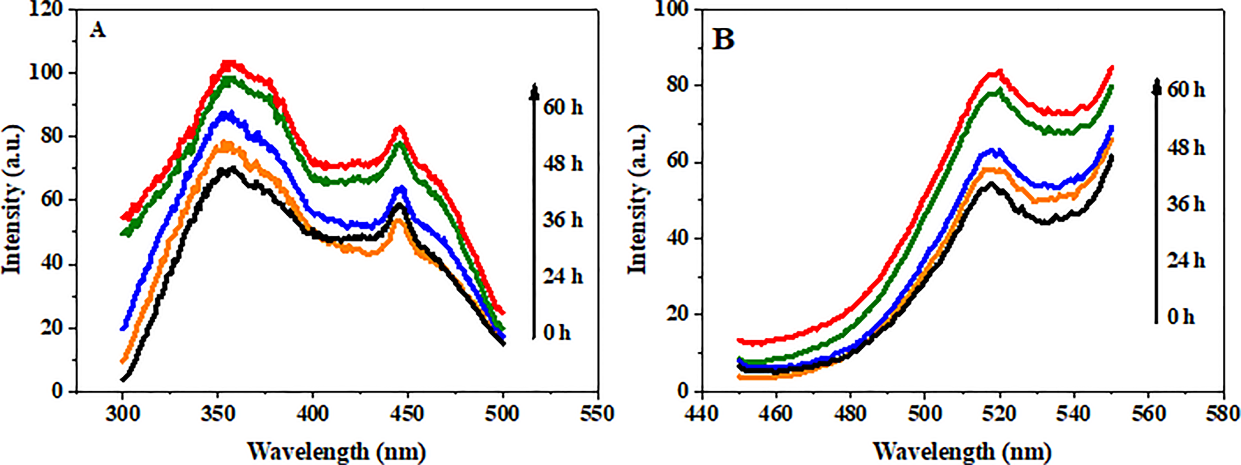
**

**Figure S3.** Identification of flavins secreted by *G. sulfurreducens*. Emission (A) and excitation (B) spectra of the cell-free culture medium collected from the *G. sulfurreducens* culture after 0, 24, 36, 48 and 60 h of inoculation.

**
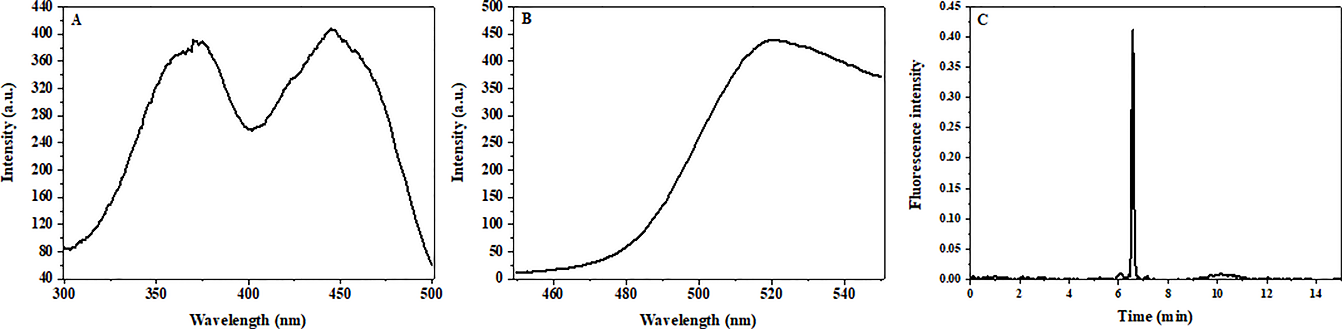
**

**Figure S4.** Identification of riboflavin secreted by *G. uraniireducens* when ferrihydrite was electron acceptor. Excitation (A) and emission (B) spectra and (C) Liquid chromatography pattern of cell-free culture medium collected from *G. uraniireducens*.

**Table S1.** The expression of outer membrane c-type cytochromes in *G. uraniireducens* growing on anode and in ferrihydrite culture medium *^a^*

|  | **Anode Biofilm** | **Ferrihydrite**  **culture** |
| --- | --- | --- |
| **Gura_0092** | **+** | **-** |
| **Gura_0500** | **+** | **+** |
| **Gura_2997** | **+** | **-** |
| **Gura_0641** | **+** | **-** |
| **Gura_3839** | **+** | **+** |
| **Gura_1316** | **+** | **-** |
| **Gura_0994** | **-** | **+** |
| **Gura_0993** | **+** | **+** |
| **Gura_3284** | **-** | **+** |
| **Gura_0989** | **+** | **+** |
| **Gura_3135** | **+** | **-** |
| **Gura_3843** | **+** | **-** |
| **Gura_4121** | **+** | **+** |

*^a^* +, identified or present. -, not identified or present.
